# The role of regulatory policies in organizational culture: Insights from the education industry

**DOI:** 10.1371/journal.pone.0299848

**Published:** 2024-05-15

**Authors:** Bin Wang, Aslan Amat Senin, Ungku NorulKamar Ungku Ahmad

**Affiliations:** Faculty of Management, Department of Management & Technology, Universiti Teknologi Malaysia, Johor Baharu, Johor, Malaysia; Babes-Bolyai University, Cluj-Napoca, ROMANIA

## Abstract

On February 26, 2018 and July 24, 2021, the Chinese government respectively issued two significant regulatory policies to address the problems caused by off-campus training institutions in terms of students’ extra-curricular and family financial burdens. These policies have had a tremendous and far-reaching impact on the off-campus training industry in China. With the help of these two events, we explored the role of industry-level regulatory policies in shaping and forming organizational culture. This paper adopts a text analysis method, combined with the dimensions of the Denison Organizational Culture Survey (DOCS) and MAXQDA 18 software, to obtain data on corporate culture. Then, the approaches of regression discontinuity in time (RDiT) and regression discontinuity (RD) designs with multiple cutoffs are used to estimate the policy treatment effect. This empirical research suggests that regulatory policies have a significant impact on corporate culture. Moreover, regulatory policies of varying degrees of strictness have differential effects on different dimensions of corporate culture. The research findings contribute to the theories of corporate culture and can guide enterprises to evaluate the impact of policies on corporate culture more clearly, thereby enabling them to make wiser operation decisions.

## 1. Introduction

Jensen et al. [1]’s *Theory of the Firm*: *Managerial Behavior*, *Agency Costs and Ownership Structure* has enjoyed great popularity for over the past 40 years. Nonetheless, it is believed that this theory and framework is not capable of explaining the entire organizational behavior and organizational or corporate culture can be a new paradigm instead [2], even though other means of corporate governance are considered to be significant for organizational achievements as well, like board of directors and managerial incentives [3], human and relational capital [4], blockchain-based smart governance systems [5], and artificial intelligence-based decision-making algorithms [6]. Indeed, over the past years, a flood of studies and literature have been produced to account for the various relations between organizational effectiveness and corporate culture [7–10]. However, as Gorton et al. [2] asked in puzzlement, what role industry-level regulatory policies play in shaping the organizational culture. Indeed, to our knowledge, there has been no literature fully confirming the relation between them empirically.

To address the issue, this paper takes the China’s off-campus training industry as the research object to explore the effect of regulatory policies on corporate culture as the industry experienced two significant regulatory policy changes in the year of 2018 and 2021. Thus, by observing and analyzing the evolution of organizational culture before and after these two policy changes, we can fully understand the impact of regulatory policies on organizational culture and summarize the role of regulatory policies in shaping corporate culture.

Specifically, three main aspects are explored in the following. First, applying the framework of the Denison Organizational Culture Survey (DOCS) developed by Denison and his colleagues [11,12], we analyze the impact of the same regulatory policy on different dimensions of corporate culture, and meanwhile examine the dissimilarities in the effect of different types of regulatory policies on the same dimension of corporate culture. Little extant literature has touched on this area, while regulatory policies appear to have a significant treatment effect on corporate culture. Second, the regression discontinuity (RD) designs are adopted to identify whether there are statistically significant changes in corporate culture, including sharp RD, regression discontinuity in time (RDiT), and RD with multiple cutoffs, which have infrequently been applied in previous researches on corporate culture, where OLS regressions were often carried out [13–15]. Finally, with the aid of MAXQDA 18 software incorporating advanced computer-assisted algorithms for tokenization, sentence segmentation and word frequency techniques [16], a corpus of 4000 news reports (around 4 million words) is employed to obtain data on four dimensions of corporate culture. By comparison, survey questionnaires have often been regarded as a measurement approach in previous research on corporate culture [17], for instance, the classic DOCS [12], the Organizational Culture Assessment Instrument (OCAI) [18] and so forth.

In conclusion, this study contributes to the understanding of the effects of regulatory policies on the different dimensions of corporate culture. Additionally, the RD designs performed in the empirical analysis innovates the research methods for corporate culture. Last, the leading text analysis software is utilized to extract data on corporate culture from news reports, which provides a unique and valuable perspective.

## 2. Research context and conceptual background

### 2.1 Public policy types

There are a range of schemes to categorize public policies. According to the impact of public policies on society, Lowi [19] divided them into three categories: distributive, regulatory and redistributive. Considering the distinct governing resources employed by the government, Hood [20] came up with a useful “NATO” model, which refers to a central policy actor (“nodality or N”), legal powers (“authority or A”), money (“treasure or T”), and available organizations (“organization or O”) [21]. On account of the social construction and political power of the target citizens, Schneider et al. [22] separated policy tools into five types: capacity-building, symbolic and hortatory, authoritative, information and dominant tools. Also, encouraging and restrictive policies were mentioned by some scholars [23–26].

In general, as Birkland [27] states, each policy classification dimension has certain advantages, disadvantages and scope of application, and there is only the most appropriate taxonomy rather than the best.

Therefore, combining the policy taxonomy with the characteristics of China’s regulatory policies towards off-campus training institutions, this study views regulatory policies as three categories from a two-dimensional perspective: encouraging, normative and restrictive policies. To put it simply, the two dimensions count on “whether large-scale growth is permitted” (whether enterprises can be profitable and use capital to expand their business) and “the number of regulatory policies” (the policies formulated by the regulatory authorities for enterprises), the purpose of which is to identify the degree of strictness of regulation.

The categories of public policies on off-campus training institutions in China are shown in Table 1. First, encouraging policies allow large-scale development of corporations, and the number of normative policies is small. For example, in this study, the stage one (from February 1993 to February 2018) of off-campus training market in China. Second, large-scale development is also supported by normative policies while the number of regulatory policies issued by the government rises dramatically, such as the stage two (from February 2018 to July 2021). Finally, restrictive policies prohibit large-scale development and for-profit operation of organizations, where meanwhile the number of regulatory policies is numerous like normative policies, as in the case of the stage three (from July 2021 to January 2022).

**Table 1:** The categories of public policies on off-campus training institutions in China.

	Stage	Whether large-scale growth is permitted	The number of regulatory policies
Encouraging policies	One	Yes	Few
Normative policies	Two	Yes	Many
Restrictive policies	Three	No	Many

### 2.2 Public policy in education

Stage one: from February 1993 to February 2018

Aiming to lessen the imbalance between supply and demand of education, and to introduce more social forces to China’s educational undertakings, the Chinese government promulgated two programmatic documents on education in 1985 and 1993 respectively—*the Decision of the Central Committee of the Communist Party of China on the Reform of the Educational Structure* and *Program for Education Reform and Development in China* [28].

It was clearly pointed out that the state should change the pattern of only government running schools into an educational system with joint efforts from both the government and all sectors of society. Furthermore, as for the social groups and individual citizens launching their careers in education in accordance with the law, the state adopted the policy of active encouragement, vigorous support, and correct guidance. Since then, the marketization and industrialization of education has begun to germinate in China. In other words, education has also become a commodity with market transaction attributes.

On the basis of this public policy, non-state-owned schools, like off-campus training institutions, began to engage in the education industry. In the next 30 years or so, they achieved vigorous development with more than 10 listed education-related corporations from China in the United States alone as of 2018, including well-known training institutions, such as New Oriental (EDU), Tomorrow Advancing Life (TAL) and the like.

Stage two: from February 2018 to July 2021

After nearly 30 years of development, off-campus training institutions made a significant achievement, playing a role in supplementing public schools. However, the “test-taking”-oriented training caused the excessive burden of extracurricular learning for primary and secondary school students, increasing the financial burden on students’ families. Meanwhile, due to the lack of supervision, it is no wonder there were potential safety risks, no relevant permits and licenses, runaway bosses, etc. in the whole education industry, which led to serious repercussions among the public.

Therefore, on February 26, 2018, with the aim of regulating the off-campus training industry, the Chinese government issued *Opinions on Regulating the Development of Off-campus Training Institutions*, governing the industry for the first time at national level [29].

Those who violate the relevant law and discipline may encounter a severe punishment. For instance, on June 1, 2021, 15 off-campus training corporations was imposed a total fine of 36.5 million yuan for market violations by the State Administration for Market Regulation [30].

Stage three: from July 2021 to January 2022

The Chinese government’s original intention of involving other sectors of society in running schools was to make up for the imbalance between supply and demand of education existing since the founding of the People’s Republic of China. On the one hand, off-campus training did offset this disequilibrium of educational resources to a certain extent. On the other hand, a family economic foundation was required to sign up for private after-school training courses. Thus, compared with students participating in off-campus training, children with weak family economies or from remote mountainous areas were bound to get left behind as a consequence of their failing to get access to extra or premium educational opportunities, which in turn caused a new imbalance in educational resources.

In light of such negative effects, on July 24, 2021, the Chinese government further promulgated a stricter regulatory policy entitled *Opinions on Further Reducing the Burden of Homework and Off-campus Training for Students in the Compulsory Education Stage* or referred to as *Double Reduction* [31].

The policy sets a series of significant restrictions on off-campus training institutions. For example, curriculum subject-tutoring institutions shall be uniformly registered as non-profit institutions; these institutions are not allowed to go public for financing; listed companies are not allowed to invest in the institutions; capitalization operations are strictly prohibited. In conclusion, this restrictive policy has led to the transformation, cancellation or even delisting of a large number of off-campus training institutions.

### 2.3 Content analysis

Content analysis, also referred to as textual analysis in a narrow sense, is a research technique, through which valid arguments can be inferred based on texts, works of art, images, symbols and so forth [32]. Not only can content analysis be adopted for qualitative analysis, but texts, works of art, images, etc. can be converted into numerical data for quantitative analysis as well [33,34]. It has been widely used by researchers in social science research. For instance, counting on the positive and negative expressions in certain texts [35–40], many scholars apply qualitative content analysis to evaluate article tone or sentiment, online communications and commitment to brand [41], gender bias in video game dialogue [42], the effect of social media influencers on travel decisions [43]. Moreover, quantitative content analysis is exploited to investigate business ethics [44], portrayals of different genders on primetime television [45], foreign news stories and sourcing practices [46], the credibility and reliability of corporate social responsibility reports [47], dialogic relationships on social media [48] etc.

Similar to our study, there are some studies where the method of quantitative content analysis is employed to measure corporate culture. For example, content analysis of annual reports of listed companies is conducted to gauge the dimensions of organizational culture [49–51]. Comparable to corporate annual reports, 10-K filings of listed firms are used to measure corporate culture [52–55]. Nevertheless, it is possible that annual reports of listed companies may exhibit significant similarity among different enterprises as a result of the desire to meet investors’ expectations [49]. Consequently, other types of texts are also considered for content analysis to measure organizational culture. For instance, Li et al. [56] analyze earnings call transcripts to measure five dimensions of organizational culture. Likewise, Liu et al. [57] measure organizational teamwork culture with earnings call transcripts. Furthermore, statements about core values on corporate official websites [58,59] and employee reviews as a type of texts originating from within the organization [60] are adopted to measure corporate culture.

Additionally, of equal importance is selecting an appropriate software to process these characters. With the advance of modern computer technology, several distinct means can now be used for content analysis, such as R language, Python scripts and computer software. And the common computer software packages are ATLAS.ti, QSR NVIVO and MAXQDA [61]. MAXQDA, where 15 different languages can be selected for its interface including Chinese language, is able to extract quantitative data in social science research based on contents like audio or video recordings, web pages, texts and the like, which makes our text analysis possible [16]. More importantly, MAXDictio function of MAXQDA 18 software provides custom dictionaries, which makes it likely to investigate the frequency of the key words concerning organizational culture in our study.

### 2.4 The Denison organizational culture survey (DOCS)

Plenty of scholars has defined organizational or corporate culture from various perspectives [62–65]. Based on almost the identical assumption with Cameron et al. [18] concerning the circumstances where corporations tend to be faced with two main types of options, that is, “external or internal” and “flexibility or stability”, Denison et al. [12] deduced the Denison organizational culture model and validated it through abundant samples, the four different organizational culture types of which are described as follows.

First, the internality-flexibility or involvement culture is involved with three aspects: empowerment, team orientation, and capability development. Empowerment refers to employees’ being authorized to attend to their own assignments, where naturally a sense of autonomy, ownership, duty and obligation is generated. Furthermore, team orientation means teamwork is emphasized, all staff members are of responsibility, tasks are expected to be accomplished on the basis of a collaborative group. Finally, capability development indicates that in an effort to obtain and remain business competitiveness, investment in workers’ comprehensive skills and capabilities is devoted.

Second, the internality-stability or consistency culture has something to do with core values, agreement, coordination and integration, which suggests employees are bound together with a set of shared core values. In this case, achieving agreement at both the implicit and explicit level is much easier. More importantly, under the effects of shared values, coordinating among different departments of the companies and integrating into the teamwork are smoother than ever before, contributing to completing the anticipated goals.

Third, the externality-flexibility or adaptability culture is concerned with creating change, customer focus and organizational learning. As a result of focusing on adaptative mechanism, this sort of organization is capable of reacting quickly, managing rapidly changing business background and looking forward to taking challenges. Certainly, customers rank first in these organizations, where customers’ needs, preference and habits are valued and satisfied especially on the occasion of highly competitive business settings. On this account, improving knowledge, competence and expertise is of great significance.

Finally, the externality-stability or mission culture is with respect to strategic direction and intent, goals and objectives, as well as vision. “Mission” is the center of organizational operation owing to its resulting in the organizational direction and intention in strategy, subsequently assisting in setting long-term or short-term objectives, shaping and modifying staff members’ behavior for an envisioned attractive future.

Notably, organizational culture can be measured in a variety of ways. What has been employed widely are the OCAI [18], the DOCS [12], Hofstede’s adopting a three-phase design to determine the dimensions of organizational culture [66] and the Organizational Culture Profile (OCP) [67].

As for this paper, the DOCS is selected [12] because its dimensions are relatively clear, and its internal and external focus, flexibility and stability, and subdivision indicators under the main items can well reflect the impact of policies on corporate culture, showing good compatibility with this study. As stated above, the DOCS is summarized by four cultural traits that characterize an organization’s culture: involvement (internal and flexible focus), consistency (internal and stable focus), adaptability (external and flexible focus), and mission (external and stable focus). These traits are represented on the two ends of a horizontal axis ranging from stable to flexible and on a vertical axis ranging from internal to external. Each of these cultural traits is measured by three indices. For instance, the three indices for the involvement trait are empowerment, team orientation, and capability development. Each index is further measured by five Likert-type items that adopt a 5-point response scale ranging from 1 (strongly disagree) to 5 (strongly agree).

Certainly, since the DOCS is a set of unified indicators for all enterprises, certain adjustments to the indices have been made in view of the characteristics of the off-campus training industry in China to improve adaptability. First, since the standardized operation of the organizations is one of the main goals of the normative and restrictive policies in China, adding keywords related to standardization can help detect the impact of the policies on organizational culture. However, there are no indices about normativeness in the DOCS. By comparison, it is found that the normativeness of an organization falls under the category of internal and stable focus and is most closely related to core values in the DOCS. Therefore, synonyms related to normativeness are added to the core values index. Second, under the capability development index, the keyword “teacher training” is added, which is similar to “employee training”, as a significant portion of employees in the off-campus training institutions are teachers. Additionally, under the goals and objectives index, the keywords such as “financing, listing, capital” are included since the off-campus training institutions in China were in a period of rapid development before the normative and restrictive policies, and financing and listing were widely discussed in the industry. These keywords can also be used to verify whether the policies have an impact on organizational culture. Finally, under the creating change index, the keywords including “cancel, slash, eliminate, outlaw, job transfer, change profession, transform” are added as well. For one thing, these keywords align with the media’s reporting habits on the off-campus training institutions in China. For another thing, the need for profound changes within the off-campus training institutions is implied in these keywords. With all these in mind, the final version of organizational culture survey of off-campus training institutions in China adapted from the DOCS is shown in the S1 Appendix.

### 2.5 Research assumptions

Taking the DOCS into account, the internality-stability or consistency culture involves three key indicators: core values, agreement, as well as coordination and integration, where an internal and stable focus is reflected [12,68]. In the meantime, it can be reasonably concluded that regulatory policies formulated by the government point to the internality of organizations, and guided by the regulatory policies, enterprises conducting standardized operation contributes to enhancing their own stability. Thus, these three key indicators along with their corresponding synonyms reflecting normative operation are integrated into the internality-stability culture, which is shown in the S1 Appendix.

Meanwhile, in view of the above classification of public policies (see Table 1), if the normative policies are chosen as the regulatory policies, the government will adopt plenty of regulatory policies to supervise the business behavior of corporations. In addition, as with the normative policies, there are loads of regulatory policies when the restrictive policies are the top priority. Accordingly, hypotheses 1 and 2 are proposed.

H1: Normative policies have a significant positive effect on the internality-stability culture of enterprises.
H2: Restrictive policies have a significant positive effect on the internality-stability culture of enterprises.

In light of the DOCS, the internality-flexibility or involvement culture, concentrating on the internal dynamics and flexibility of the organization, relies on three key indicators: empowerment, team orientation and capability development, the items of which are employee engagement, teamwork, skill training, etc. [12,68]. Generally speaking, as long as corporations aim to survive and develop, no matter how the external environment changes, the flexibility of the internal operation of enterprises will maintain a certain intensity at any time, which will be less affected by external regulatory policies. For instance, seven determinants of employee engagement like work environment, compensation, workplace well-being and so forth, no external regulatory policies are mentioned [69]. The same situation occurs in the empowerment aspect [70,71]. Accordingly, hypotheses 3 and 4 are put forward.

H3: Normative policies do not significantly affect the internality-flexibility culture of enterprises.
H4: Restrictive policies do not significantly affect the internality-flexibility culture of enterprises.

Furthermore, the externality-stability or mission culture involves three core indicators: vision, goals and objectives, as well as strategic direction and intent, which presents a concentration external to enterprises and centered on stability [12,68]. Generally speaking, complying with regulatory policies might increase the operating costs and difficulties of profitability, and weaken the willingness of capital investment and the confidence of enterprises in the future prospects of the industry [72]. Moreover, when regulatory policies are so strict that they limit the scale of a company, the vision, goals and strategic direction indicators of the company will be further weakened.

The treatment effect of these two policies, however, is likely to be divergent. The main reason consists in the different purpose of the two regulatory policies. Fundamentally, the normative policies do not set restrictions on massive development and profitability for private tutoring institutions (see Table 1). Thus, the weakened willingness and confidence maybe just exist for a short period of time, which will be strengthened once again when the potential intention of the normative policies is recognized by the intelligent leaders and managers in the industry. The restrictive policies, nevertheless, are quite the opposite, which do set restrictions on the large-scale growth and profitability of the off-campus training institutions (see Table 1) and probably result in continuously weakened motivation and initiative. Thus, hypotheses 5 and 6 are proposed.

H5: Normative policies have a significant negative effect on the externality-stability culture of enterprises temporarily.
H6: Restrictive policies have a significant negative effect on the externality-stability culture of enterprises.

Finally, the externality-flexibility or adaptability culture is concerning three core indicators: organizational learning, customer focus, and creating change, which involves in externality and flexibility in an organization [12,68]. Logically, normative policies do not impose requirements on organizational learning, customer orientation or organizational change, mainly concentrating on corporate internal normativeness [29,73]. Restrictive policies, nevertheless, strictly confine corporate financing, profitability and scale [31], which will lead enterprises to actively seek business innovation and corporate change to adapt to severe policy adjustments. Hence, hypotheses 7 and 8 are put forward.

H7: Normative policies do not significantly affect the externality-flexibility culture of enterprises.
H8: Restrictive policies have a significant positive effect on the externality-flexibility culture of enterprises.

## 3 Empirical strategy

### 3.1 Data sources and sample screening

The research subject of this study focuses on the off-campus training institutions in China. There are two periods of the study, the first of which is from August 14, 2017 to August 19, 2018 to estimate the treatment effect of the normative policies and the second is from January 9, 2021 to January 21, 2022 to estimate the treatment effect of the restrictive policies. Either of the periods contains 50 sets of weekly data. Also, for weeks whose data collection are terminated due to public holidays, including National Day, Labor Day, Spring Festival, etc., the approach of eliminating these weekly data is adopted as these data miss completely at random (MCAR) [74–76].

In the course of our collecting data, 3 weekly data in the first period and 4 in the second were not available out of public holidays. However, since neither of them accounted for more than 10% of the whole weekly data as well, they were removed and replaced by the according following weeks.

The options for collecting data concerning corporate culture in the form of texts generally include annual reports, official websites of different corporations and mass media reports [32].

First, annual reports. As a result of the small overall number of listed companies of off-campus training institutions, and the existence of U.S. listed companies which is not suitable for Chinese text analysis, the number of corporate annual reports reflecting the organizational culture is very limited. Moreover, the reporting time of annual reports of enterprises is on an annual basis, which does not match the time cutoff studied in this paper, so they cannot be used as a source of sample data.

Second, official websites. Out of the needs of corporate operation, the official websites of most off-campus tutoring institutions are used for signing up for courses. Consequently, the number of official websites that can reveal the changes in corporate culture is not enough as well.

Finally, media coverage. Since corporate culture can be disseminated through the media, it is feasible to conduct text analysis with authoritative media reports to measure corporate culture [77]. There are a great number of news reports about off-campus training institutions, which is the crucial source of our sample.

The data of corporate culture was obtained manually in this study through the database of China Digital Library developed by Beijing Founder Apabi Technology Co., Ltd, which contains various newspaper resources at all levels in China. Approximately 500 official news media were retrieved. After making the most of the database to accomplish multiple data comparisons, it was found that an average of 40 weekly media report texts on the theme of off-campus training institutions could be retrieved. Eventually, 2000 texts were attained for 50 weeks in either of the study period, which signifies a total of 4000 texts for content analysis.

### 3.2 The measurement of organizational culture

Similar to Fiordelisi et al. [49], the approach of measuring corporate culture in this study is that the occurrence of a certain keyword in the sample represents some corresponding corporate culture of an organization, and that the change in the frequency of keywords in the sample also represents a corresponding alteration in organizational culture. The specific steps are as follows.

First, the core keywords are distilled according to the DOCS [12]. Second, the synonyms of these keywords are ascertained by referencing dictionaries like Xinhua Dictionary, Modern Chinese Dictionary, Oxford Dictionary, Longman Dictionary, Collins Synonyms Dictionary and so forth to ensure a comprehensive measurement of the strength of the corporate culture represented by a certain keyword. A complete listing of these items is included in the S1 Appendix. Here is an example of the first two steps. One of the items in the DOCS is “We have a shared vision of what the organization will be like in the future.” The core keyword “vision” in the sentence is distilled and the synonyms like “foresight” and “prospect” in Chinese are found out according to the dictionaries. Third, the custom dictionary is established in the MAXQDA 18 software as stated by the bag of words in the S1 Appendix. Fourth, the stop words are removed. To avoid some meaningless words affecting the word frequency of the keywords, some meaningless numbers, letters and words are removed, such as date, daily, evening news, etc., on the basis of referring to the Baidu stop word list and considering the characteristics of the sample of this study, so as to guarantee the comparability between the number of the key words and the total number of the words in the sample. What’s more, the influence of negative words has also been eliminated in this study. For instance, the keyword “financing” appears in “financing” and “prohibit financing”, but the implication behind them is completely opposite. Fifth, the content analysis of 4000 texts containing nearly 4 million Chinese characters of media coverage of China’s off-campus training institutions is performed in the MAXQDA 18 software, and the sum of the frequencies of all the synonyms of a certain index is considered the value of that dimension of organizational culture. Take the index “vision” in the S1 Appendix for example, the sum of the frequencies of all the synonyms including “vision, foresight, prospect, long-term viewpoint, forethought, prosperity, booming” is viewed as the value of the index “vision”. Naturally, the value of the trait “externality-stability or mission culture” is equal to the sum of the values of the three indices “vision, goals and objectives, and strategic direction and intent”.

### 3.3 Regression discontinuity in time (RDiT) and cumulative multi-cutoff regression discontinuity

There are a growing number of policy scientists beginning to adopt RD designs to recognize causal effects [78]. Applying the RD designs, researchers divide the observed data into two groups: one group consists of observations near the cutoff or threshold that receive a certain treatment (e.g., a policy intervention), and the other group is made up of observations around the threshold which do not receive the treatment. By carrying out a local polynomial regression, the treatment effect of an intervention can be estimated [79]. The RD designs are considered quasi-experimental designs since the analytical data close to the cutoff are employed, which to some extent ensures that other determinants around the cutoff remain relatively stable. This helps to better control confounding factors, allowing for attributing the observed treatment effect to the intervention rather than other factors and thus making unbiased estimation possible [80,81]. As a consequence of the advantage of the RD, it has been applied in various fields of social sciences, including economics, policy science, education, labor markets, health, environment, and so forth [78,80].

RD designs can be divided into two categories: “sharp” and “fuzzy” RD designs, the difference between which consists in whether the probability of treatment jumps from 0 to 1 at the cutoff or not [80]. Specifically, in sharp RD cases, each individual of the sample is bound to be treated at the cutoff where there is an according treatment effect for each individual, while in fuzzy RD cases, the individuals of the sample are just likely to be treated at the cutoff where there is not necessarily a corresponding treatment effect for each individual.

In light of the aforementioned categories of public policies, all the off-campus training institutions in China definitely received the according treatment effect from the normative and restrictive policies, which indicates the probability of treatment jumps from 0 to 1 at the cutoff. Thus, the sharp RD is used to estimate the treatment effects.

Furthermore, it is noteworthy that time acts as the running variable in the study. Consequently, the characteristics of regression discontinuity in time (RDiT) which are distinct from the conventional RD should be taken into consideration [82]. The details are as follows.

First, the McCrary [83]’s density test that is utilized to check whether there is manipulation of the running variable on either side of the cutoff is not applicable to RDiT. Thus, the density test is not employed as a result of its applicability.

Second, in the RDiT, to get sufficient number of observations flanking the cutoff, the data far from the cutoff may be chosen to complete an estimation. This, however, runs counter to the underlying advantage of RD designs that rely on the data as close as possible to the threshold to accomplish a regression like the quasi-experimental framework. On this account, to reduce the harmful impact from the remote data and meanwhile maintain a specific amount of them, 25 observations or weeks on either side of the cutoff, 50 in total, are employed to carry out our RDiT assessment.

In addition to RDiT, RD with multiple cutoffs has also appeared in this study, which consists of three different types: non-cumulative multiple cutoffs, cumulative multiple cutoffs, and multiple scores [84,85]. Specifically, RD with cumulative multiple cutoffs refers to there being at least two cutoffs at the different values of the running variable. In this case, individuals receive treatment 1 if *X* (the independent variable)<*c*_1_ (the value at the first cutoff), treatment 2 if *c*_1_≤*X*<*c*_2_ (the value at the second cutoff), and the like, until the last treatment value at *X*≥*c*_J_ (the value at the Jth cutoff). With regard to the off-campus training institutions in China, cumulative multiple cutoffs occur at hypothesis 5 (H5), where there should be two cutoffs. The first cutoff lies at the week value of the government promulgating the normative policies and the second at the value of the off-campus training institutions realizing the real restrictions of the normative policies and encouraging policies beginning to take effect again.

The computer commands concerning the above RDiT and RD with multiple cutoffs are performed in the setting of Stata 16 by referencing the relative syntax from Cattaneo et al. [85] and Calonico et al. [86]. Considering the potential endogenous issue of global polynomial regression, the *rdrobust* or *rdms* command of RD designs is utilized to estimate local average treatment effect of local linear regression.

In order to verify the hypotheses proposed in this study, the following RD equation is constructed.


$${\mathrm{Culture}}_{i}=\alpha_{i}+\beta_{i}(x_{i}-c)+\gamma_{i}D+f(x_{i})+\varepsilon_{i}$$

For this equation, the dependent variable *Culture*_*i*_ stands for internality-stability culture (*Cultureis*), internality-flexibility culture (*Cultureif*), externality-stability culture (*Culturees*) or externality-flexibility culture (*Cultureef*); *x*_*i*_ is the running variable; *c* is the according week value at the cutoff; *D* is the treatment variable whose value is: *D* = 1 if *x*_*i*_≥*c* or *D* = 0 if *x*_*i*_<*c*; the function *f*(*x*_*i*_) represents a polynomial involving the cross-product terms of the running variable and the treatment variable. Table 2 displays the names, symbols and definitions of variables.

**Table 2:** Names, symbols and definitions of variables.

	Names	Symbols	Definitions
Dependent variable	Internality-stability culture	*Cultureis*	The rate of the keywords of consistency culture in the sample
	Internality-flexibility culture	*Cultureif*	The rate of the keywords of involvement culture in the sample
	Externality-stability culture	*Culturees*	The rate of the keywords of mission culture in the sample
	Externality-flexibility culture	*Cultureef*	The rate of the keywords of adaptability culture in the sample
Independent variable	The running variable	*x*_*i*_	The week value
	The treatment variable	*D*	*D* = 1 if *x*_*i*_≥*c* or *D* = 0 if *x*_*i*_<*c*

Tables 3 and 4 present the descriptive statistics of the variables, and the data in both tables are differentiated either before or after the regulatory policies [87], so that the treatment effect of the regulatory policies can be better reflected to fit the theme of this paper. According to Tables 3 and 4, *Cultureis* goes through relatively significant changes in the mean value under both regulatory policies, from 0.633% to 1.841% and from 1.673% to 3.525% respectively whereas *Cultureif* shows no significant changes under both regulatory policies. Additionally, the changes in *Culturees* are not significant in Table 3, but all the statistical values regarding *Culturees* show a significant decrease in Table 4, with the mean value dropping from 0.281% to 0.008%, the maximum value decreasing from 0.387% to 0.041%, and the minimum value dropping from 0.131% to zero. Furthermore, in Table 3, the variations of *Cultureef* are not significant, but in Table 4, the statistical values show a significant increase, with the mean rising from 0.120% to 0.508%, the maximum value increasing from 0.210% to 0.685%, and the minimum value growing from 0.063% to 0.128%. Greater changes of the data indicate more significant impacts from the regulatory policies. The data changes in Tables 3 and 4 are suggestive and generally consistent with the research hypotheses, but a more in-depth empirical analysis is needed to justify whether the changes in the values of organizational culture of China’s off-campus training institutions can indeed be attributed to the regulatory policies.

**Table 3:** Descriptive statistics of variables on the normative policies.

	Before the normative policies				After the normative policies			
	Mean (%)	Std. Dev.	Min (%)	Max (%)	Mean (%)	Std. Dev.	Min (%)	Max (%)
*Cultureis*	0.633	0.117	0.365	0.766	1.841	0.106	1.633	2.125
*Cultureif*	0.013	0.007	0.000	0.026	0.007	0.007	0.000	0.020
*Culturees*	0.501	0.071	0.362	0.609	0.440	0.132	0.191	0.655
*Cultureef*	0.257	0.059	0.157	0.344	0.218	0.045	0.132	0.318

**Table 4:** Descriptive statistics of variables on the restrictive policies.

	Before the restrictive policies				After the restrictive policies			
	Mean (%)	Std. Dev.	Min (%)	Max (%)	Mean (%)	Std. Dev.	Min (%)	Max (%)
*Cultureis*	1.673	0.383	0.951	2.192	3.525	0.405	2.968	4.352
*Cultureif*	0.005	0.008	0.000	0.032	0.004	0.008	0.000	0.035
*Culturees*	0.281	0.082	0.131	0.387	0.008	0.009	0.000	0.041
*Cultureef*	0.120	0.033	0.063	0.210	0.508	0.130	0.128	0.685

## 4 Analysis of empirical results

### 4.1 Potential manipulation

As a result of the McCrary [83]’s test of the running variable density function not being applicable to the regression discontinuity in time, theoretical analyses tend to be employed to distinguish whether there is potential manipulation of the running variable which results in endogenous grouping. By referring to Dahl et al. [88]’s analysis of potential manipulation of strategic timing of births, it is found that it is mandatory for off-campus tutoring institutions in China to obey the normative and restrictive policies, that is to say, to receive the treatment. Since they are obligatory policies, regardless of the conditions specific to each off-campus tutoring enterprise, they are bound to be treated at the exact time when the policies begin to be implemented, and the individuals from the industry do not have the autonomy to determine whether to accept the treatment or not. Furthermore, China’s central and local governments employed a wealth of conduits of news and even conducted regulatory talks with the operation leaders from the off-campus training institutions to promote the policy. In addition, tough new regulators were established to implement requirements for both online and offline tutoring schools. To say the least, if certain training institution does not obey the rules and regulations, strict penalties will put on it, as mentioned above.

Despite all these determinants, a Donut Hole approach, which means excluding the observations closest to the cutoff to avoid the potential manipulation from the individual off-campus institutions, will be employed in the later robustness check [89].

### 4.2 Graphical results

One of the advantages of RD is that visual graphics can be produced to directly display whether there are certain effects. To this end, drawing on the command *rdplot* and *twoway* in Stata 16, the fitted lines indicating the relationship between the result variable and the running variable will be exhibited below.

In Figs 1–8, the black lines in the middle represent the first week of the implementation of regulatory policies, while the other lines on either side of them are polynomial fitting based on the frequency values of keywords.

**Fig 1:**
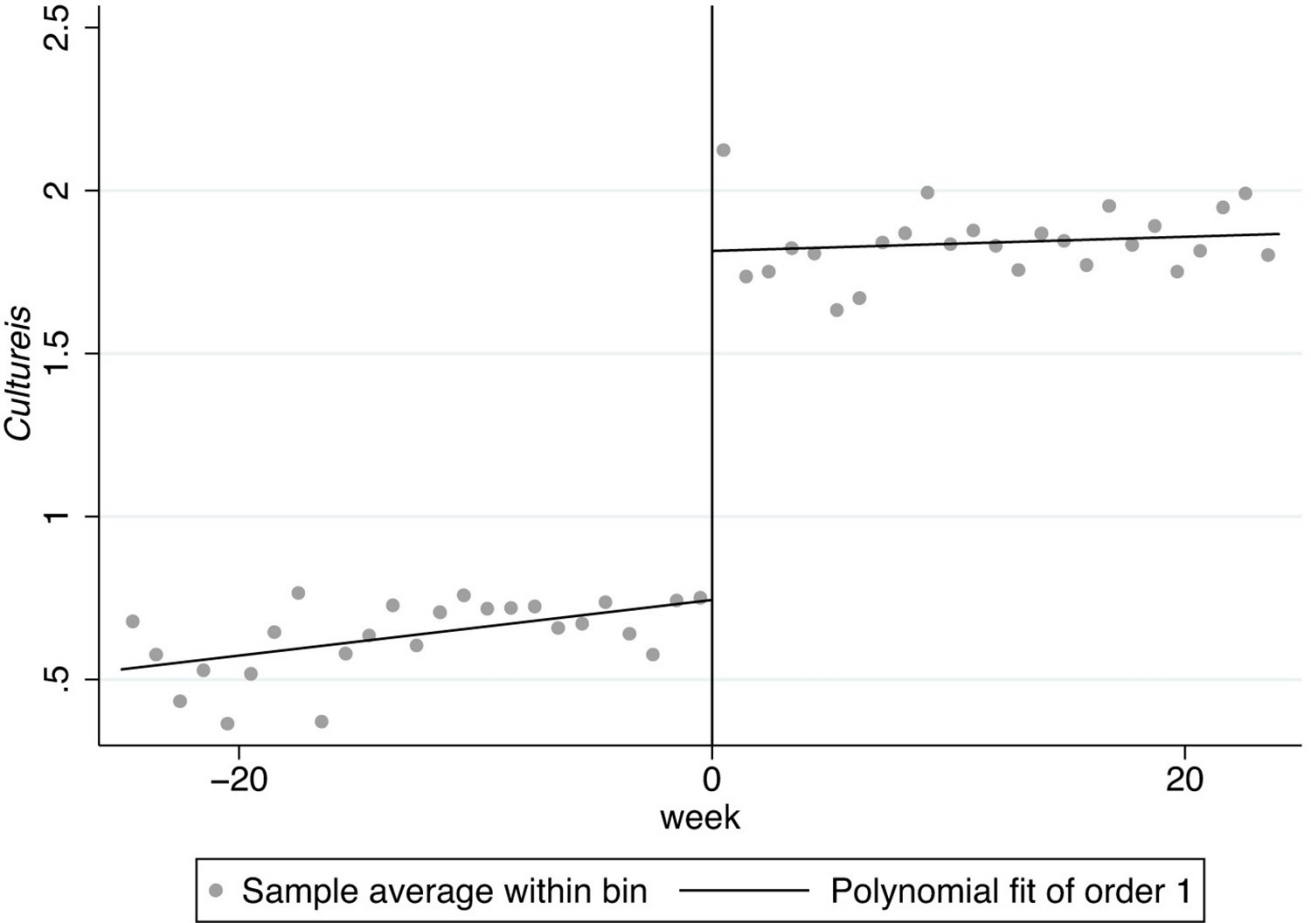
The effect of the normative policies on *Cultureis*.

**Fig 2:**
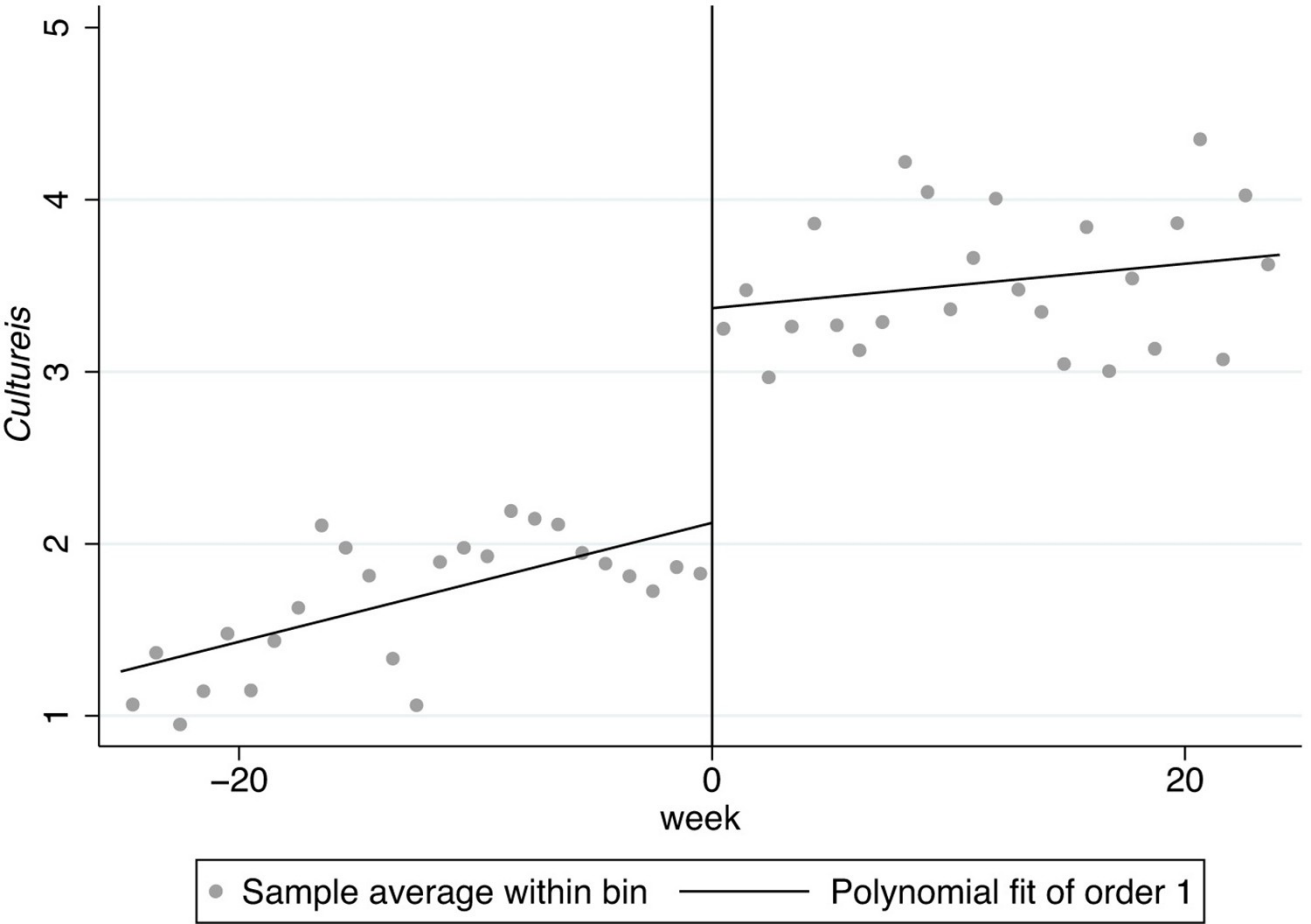
The effect of the restrictive policies on *Cultureis*.

**Fig 3:**
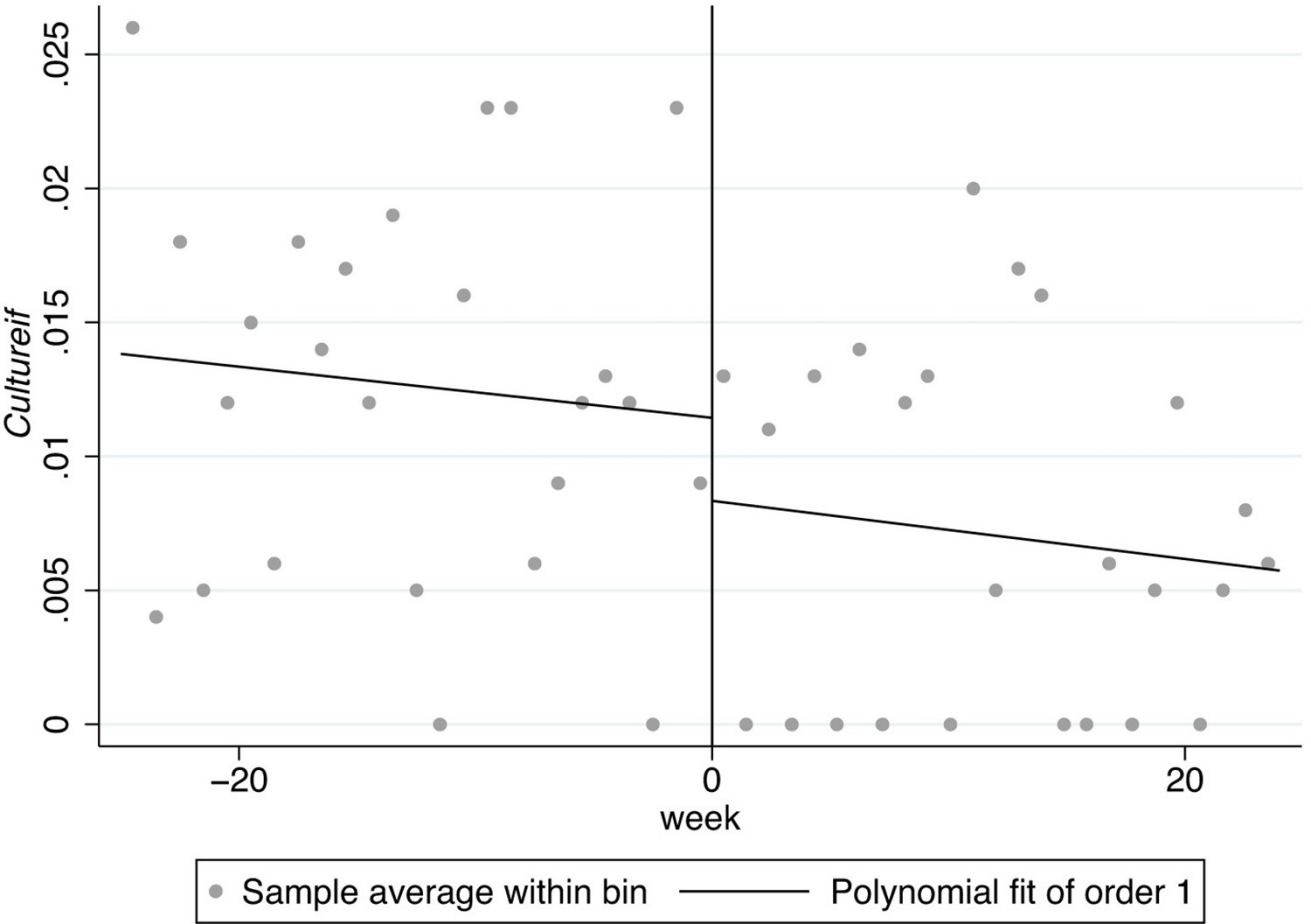
The effect of the normative policies on *Cultureif*.

**Fig 4:**
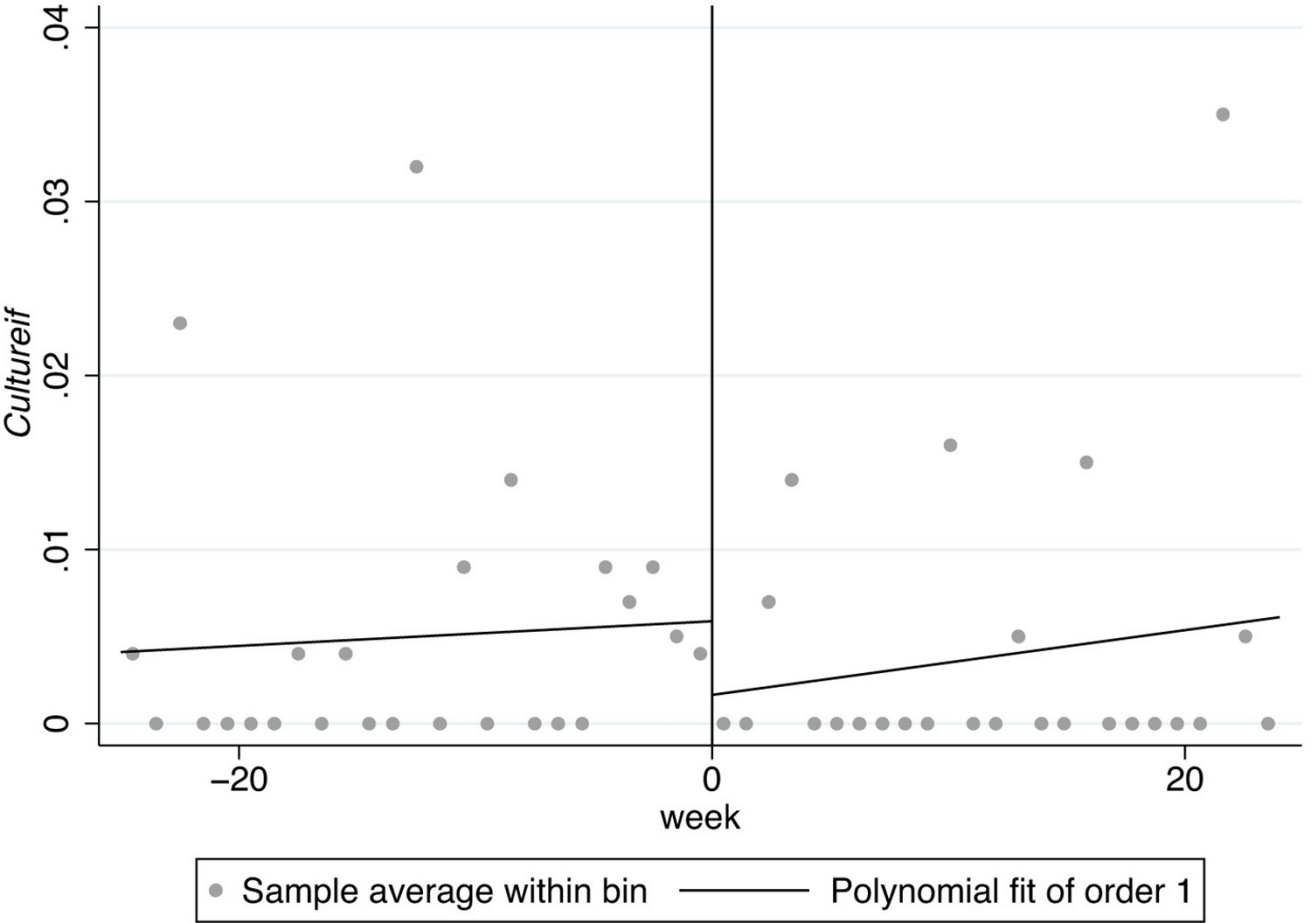
The effect of the restrictive policies on *Cultureif*.

**Fig 5:**
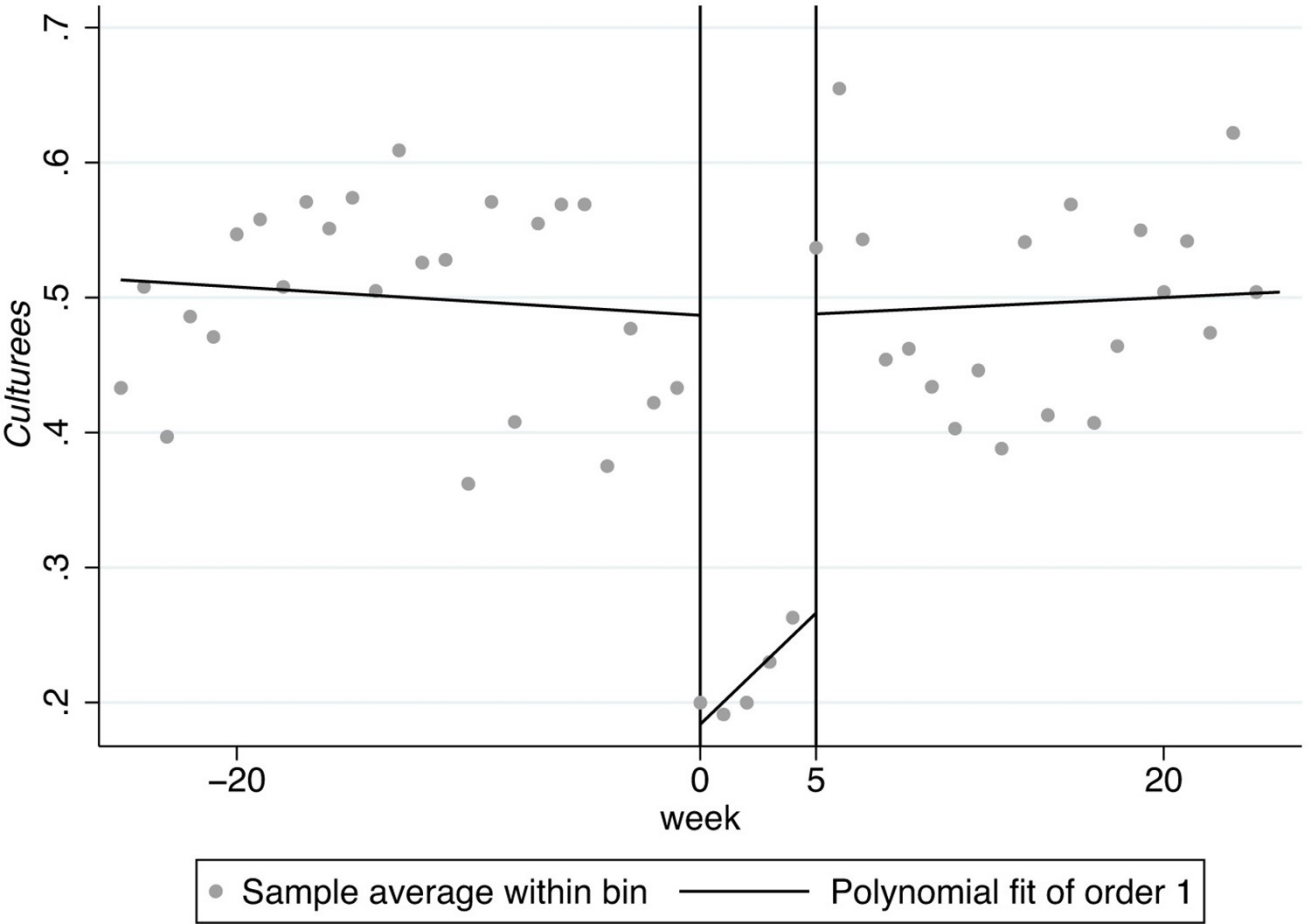
The effect of the normative policies on *Culturees*.

**Fig 6:**
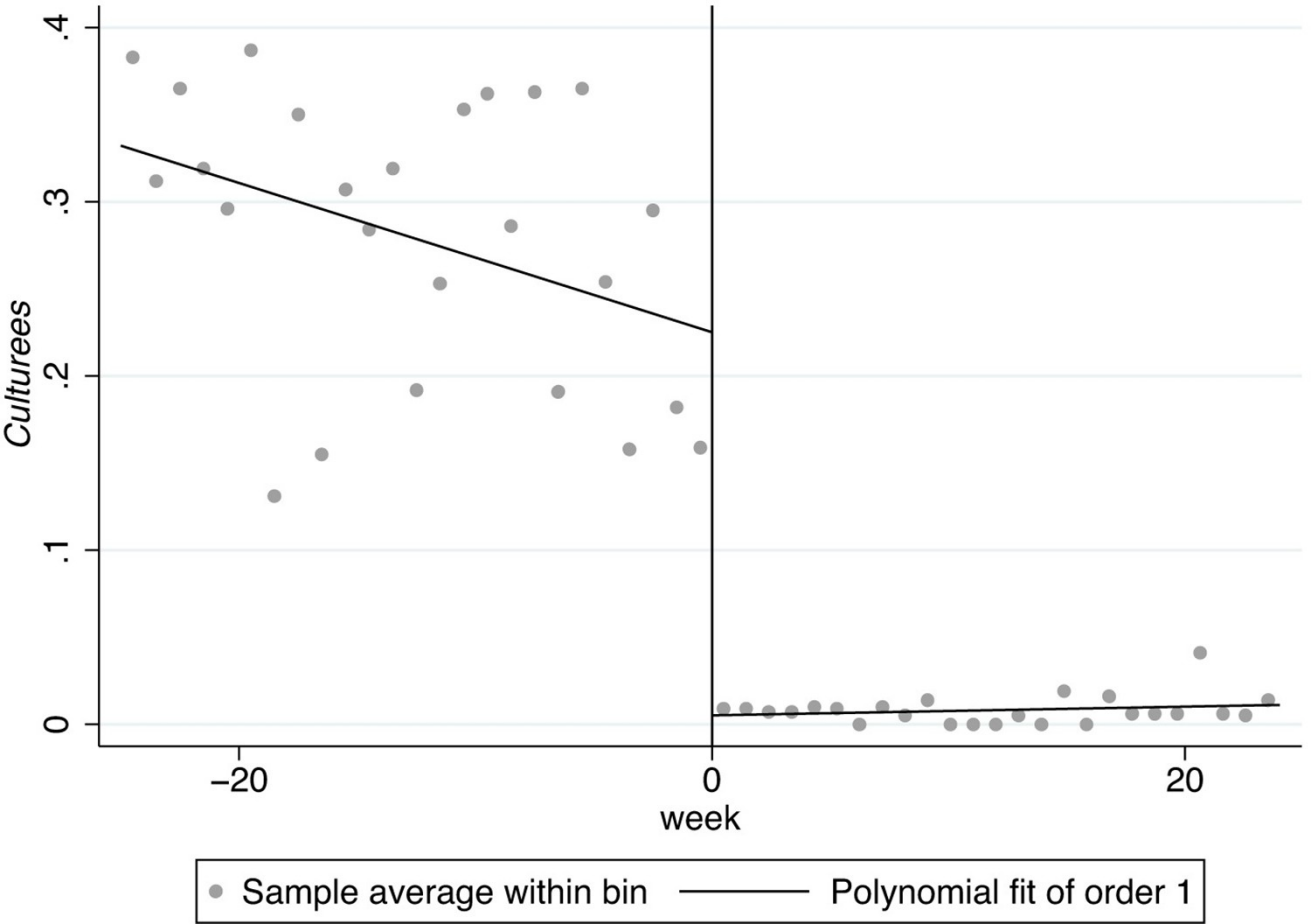
The effect of the restrictive policies on *Culturees*.

**Fig 7:**
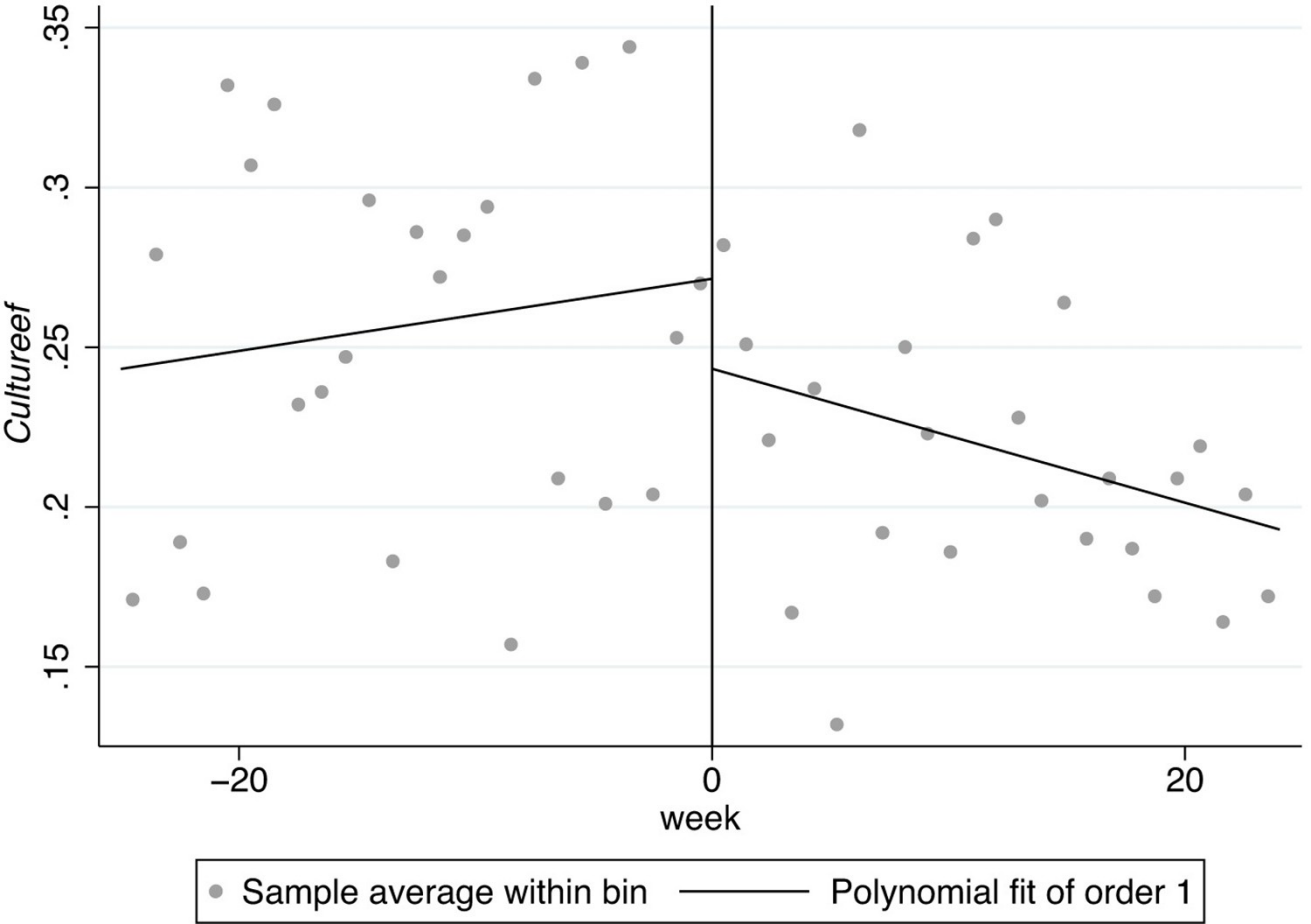
The effect of the normative policies on *Cultureef*.

**Fig 8:**
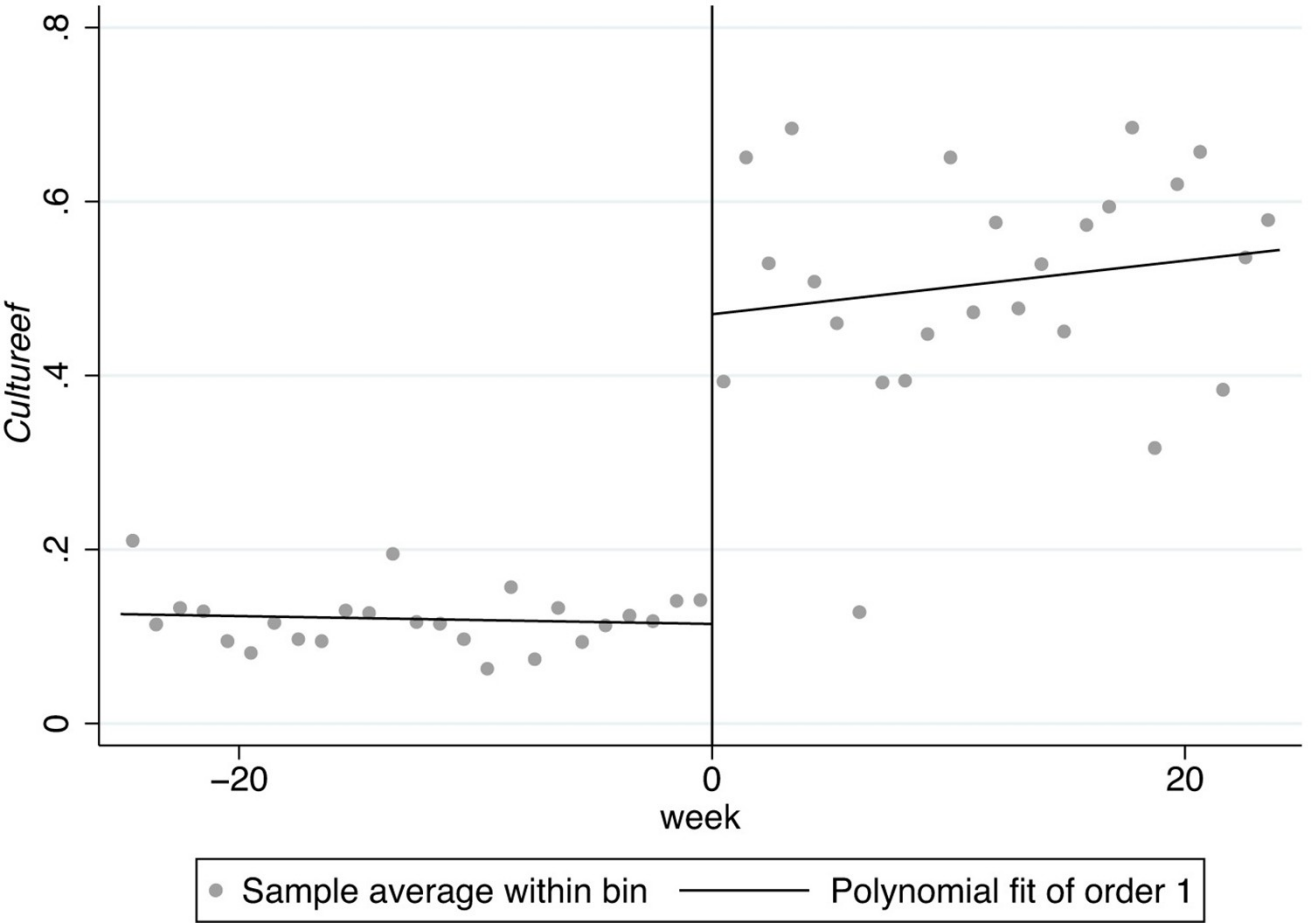
The effect of the restrictive policies on *Cultureef*.

Figs 1 and 2 display the frequency of the key words related to *Cultureis* has significant jumps at the cutoff, indicating that both types of regulatory policies may have a significant impact on internality-stability culture (*Cultureis*). Moreover, the two jumps at the cutoff move upwards, suggesting that both types of regulatory policies have a positive effect on *Cultureis*.

Based on Figs 3 and 4, the frequency of the key words related to *Cultureif* does not show significant jumps at the threshold, revealing that both types of regulatory policies may not have a significant effect on internality-flexibility culture (*Cultureif*).

Fig 5 shows the frequency of the key words related to *Culturees* significantly jumps at two thresholds, 0 and 5, which means that the normative policies may have a significant weakening effect on externality-stability culture (*Culturees*) only for a certain period, after which the externality-stability culture returns to the previous level; in Fig 6, nevertheless, the restrictive regulatory policies may have a significant weakening effect on externality-stability culture throughout.

Furthermore, there are distinguishing jumps in Figs 7 and 8, manifesting that the normative regulatory policies may not have a significant impact on externality-flexibility culture (*Cultureef*) while the restrictive regulatory policies may have a significant strengthening effect on externality-flexibility culture. A summative statement is presented in Table 5.

**Table 5:** The effects of regulatory policies on organizational culture.

	The normative policies	The restrictive policies
*Cultureis*	Significant and positive	Significant and positive
*Cultureif*	Insignificant	Insignificant
*Culturees*	Temporarily significant and negative	Significant and negative
*Cultureef*	Insignificant	Significant and positive

### 4.3 Main results

In view of the fitted patterns just showing the estimation visually, whether the regression results are statistically significant or not is the key point. Thus, we now display the regression-based estimates in detail. In general, there are four steps to conduct a local polynomial regression of RD designs [81]. First, the polynomial order *p* and the kernel function (*triangular* or *uniform*) are decided. Second, the bandwidth *h* which identifies a certain scope of observations to conduct the RD estimate is selected. Third, the classic least-squares methods to form point estimators are employed. Finally, researchers are capable of performing reasonable inference based on statistical RD parameters.

Rather than adopting the global polynomial approaches, local polynomial regression is favored since some methodological problems coming with the global polynomial can be avoided [81]. It is worth noting that high-order polynomials in regression discontinuity analysis may result in defects such as biased estimates and high sensitivity to the degree of the polynomial. So local linear or quadratic polynomials are a preferable choice [90].

Given the above steps and the order of polynomials, we decide that the local polynomial order is *p* = 1 or 2. That is to say, local linear and quadratic polynomials are applied. As for the kernel function, both the *triangular* and *rectangle* functions will be reported. Also, MSE-optimal bandwidth is applied by referring to the method of bandwidth choices [91]. Not only the MSE-optimal bandwidth, but length equal to the 2 and 3 times of it will be exhibited. Finally, the local polynomial regression results are displayed in Tables 6–10.

**Table 6:** The treatment effect of regulatory policies on *Cultureis*.

	The normative policies				The restrictive policies			
	(1)	(2)	(3)	(4)	(5)	(6)	(7)	(8)
MSE-bandwidth estimate	1.223*** (0.127)	1.218*** (0.110)	1.240*** (0.137)	1.232*** (0.123)	1.469*** (0.110)	1.366*** (0.112)	1.536*** (0.137)	1.307*** (0.131)
Bandwidth	7.449	9.129	5.080	7.091	7.107	8.696	6.511	5.355
Double MSE-bandwidth estimate	1.163*** (0.114)	1.224*** (0.140)	1.149*** (0.123)	1.197*** (0.147)	1.408*** (0.133)	1.521*** (0.174)	1.276*** (0.198)	1.530*** (0.194)
Bandwidth	14.898	18.258	10.160	14.182	14.214	17.392	13.022	10.710
Triple MSE-bandwidth estimate	1.113*** (0.087)	1.181*** (0.121)	1.128*** (0.091)	1.193*** (0.129)	1.401*** (0.137)	1.488*** (0.156)	1.470*** (0.166)	1.413*** (0.208)
Bandwidth	22.347	27.387	15.240	21.273	21.321	26.088	19.533	16.065
N	50	50	50	50	50	50	50	50
Number of texts	2000	2000	2000	2000	2000	2000	2000	2000
Polynomial	Linear	Quadratic	Linear	Quadratic	Linear	Quadratic	Linear	Quadratic
Kernel function	*triangular*	*triangular*	*uniform*	*uniform*	*triangular*	*triangular*	*uniform*	*uniform*

Standard errors clustered by *Cultureis*. Asterisks denote significance levels (* = 0.10, ** = 0.05, *** = 0.01). Each cell presents a separate estimate from a *rdrobust* analysis.

**Table 7:** The treatment effect of regulatory policies on *Cultureif*.

	The normative policies				The restrictive policies			
	(1)	(2)	(3)	(4)	(5)	(6)	(7)	(8)
MSE-bandwidth estimate	-0.004 (0.008)	-0.006 (0.009)	-0.003 (0.009)	-0.004 (0.009)	-0.003 (0.002)	-0.003 (0.003)	-0.000 (0.003)	-0.001 (0.003)
Bandwidth	5.791	10.471	5.316	7.146	6.950	9.756	5.564	8.729
Double MSE-bandwidth estimate	-0.003 (0.007)	-0.005 (0.006)	-0.001 (0.006)	-0.002 (0.006)	-0.002 (0.004)	-0.002 (0.004)	-0.003 (0.004)	-0.000 (0.005)
Bandwidth	11.582	20.942	10.632	14.292	13.900	19.512	11.128	17.458
Triple MSE-bandwidth estimate	-0.005 (0.006)	-0.005 (0.006)	-0.005 (0.006)	-0.005 (0.006)	-0.003 (0.003)	-0.002 (0.005)	-0.003 (0.004)	-0.002 (0.005)
Bandwidth	17.373	31.413	15.948	21.438	20.850	29.268	16.692	26.187
N	50	50	50	50	50	50	50	50
Number of texts	2000	2000	2000	2000	2000	2000	2000	2000
Polynomial	Linear	Quadratic	Linear	Quadratic	Linear	Quadratic	Linear	Quadratic
Kernel function	*triangular*	*triangular*	*uniform*	*uniform*	*triangular*	*triangular*	*uniform*	*uniform*

Standard errors clustered by *Cultureif*. Asterisks denote significance levels (* = 0.10, ** = 0.05, *** = 0.01). Each cell presents a separate estimate from a *rdrobust* analysis.

**Table 8:** The treatment effect of normative policies on *Culturees*.

	Cutoff at week 0				Cutoff at week 5			
	(1)	(2)	(3)	(4)	(5)	(6)	(7)	(8)
MSE-bandwidth estimate	-0.246*** (0.002)	-0.200*** (0.032)	-0.270** (0.049)	-0.229*** (0.038)	0.268*** (0.038)	0.281*** (0.061)	0.287*** (0.045)	0.283*** (0.064)
Bandwidth	2.74	14.73	10.00	15.79	3.07	9.83	10.00	7.17
Double MSE-bandwidth estimate	-0.232*** (0.033)	-0.207*** (0.029)	-0.264*** (0.035)	-0.201*** (0.033)	0.317*** (0.059)	0.265*** (0.046)	0.222*** (0.043)	0.274*** (0.051)
Bandwidth	5.48	29.46	20.000	31.58	6.14	19.66	20.00	14.34
Triple MSE-bandwidth estimate	-0.206*** (0.025)	-0.204*** (0.031)	-0.303*** (0.034)	-0.201*** (0.033)	0.315*** (0.048)	0.255*** (0.044)	0.222*** (0.043)	0.245*** (0.044)
Bandwidth	8.22	44.19	30.000	47.37	9.21	29.49	30.00	21.51
N	50	50	50	50	50	50	50	50
Number of texts	2000	2000	2000	2000	2000	2000	2000	2000
Polynomial	Linear	Quadratic	Linear	Quadratic	Linear	Quadratic	Linear	Quadratic
Kernel function	*triangular*	*triangular*	*uniform*	*uniform*	*triangular*	*triangular*	*uniform*	*uniform*

Standard errors clustered by *Culturees*. Asterisks denote significance levels (* = 0.10, ** = 0.05, *** = 0.01). Each cell presents a separate estimate from a *rdms* analysis. The results of Columns (3) and (7) derive from a customized bandwidth as a result of the failure of MSE-based local linear polynomial along with the kernel function (*uniform*) estimate.

**Table 9:** The treatment effect of restrictive policies on *Culturees*.

	The restrictive policies			
	(1)	(2)	(3)	(4)
MSE-bandwidth estimate	-0.135*** (0.021)	-0.110** (0.056)	-0.115*** (0.030)	-0.179** (0.078)
Bandwidth	7.298	7.815	6.169	6.833
Double MSE-bandwidth estimate	-0.167*** (0.031)	-0.114*** (0.031)	-0.171*** (0.037)	-0.087** (0.041)
Bandwidth	14.596	15.630	12.338	13.666
Triple MSE-bandwidth estimate	-0.208*** (0.033)	-0.148*** (0.033)	-0.221*** (0.037)	-0.159*** (0.045)
Bandwidth	21.894	23.445	18.507	20.499
N	50	50	50	50
Number of texts	2000	2000	20020	2000
Polynomial	Linear	Quadratic	Linear	Quadratic
Kernel function	*triangular*	*triangular*	*uniform*	*uniform*

Standard errors clustered by *Culturees*. Asterisks denote significance levels (* = 0.10, ** = 0.05, *** = 0.01). Each cell presents a separate estimate from a *rdrobust* analysis.

**Table 10:** The treatment effect of regulatory policies on *Cultureef*.

	The normative policies				The restrictive policies			
	(1)	(2)	(3)	(4)	(5)	(6)	(7)	(8)
MSE-bandwidth estimate	0.014 (0.025)	0.016 (0.045)	-0.017 (0.038)	0.004 (0.049)	0.386*** (0.105)	0.278*** (0.058)	0.383*** (0.097)	0.268*** (0.044)
Bandwidth	6.080	9.041	7.245	8.648	7.258	7.513	5.933	6.862
Double MSE-bandwidth estimate	-0.016 (0.032)	-0.005 (0.035)	-0.039 (0.039)	-0.019 (0.042)	0.383*** (0.067)	0.400*** (0.116)	0.369*** (0.064)	0.415*** (0.112)
Bandwidth	12.160	18.082	14.490	17.296	14.516	15.026	11.866	13.724
Triple MSE-bandwidth estimate	-0.029 (0.033)	-0.022 (0.036)	-0.011 (0.033)	-0.017 (0.040)	0.361*** (0.064)	0.398*** (0.080)	0.351*** (0.070)	0.395*** (0.076)
Bandwidth	18.240	27.123	21.735	25.944	21.774	22.539	17.799	20.586
N	50	50	50	50	50	50	50	50
Number of texts	2000	2000	2000	2000	2000	2000	2000	2000
Polynomial	Linear	Quadratic	Linear	Quadratic	Linear	Quadratic	Linear	Quadratic
Kernel function	*triangular*	*triangular*	*uniform*	*uniform*	*triangular*	*triangular*	*uniform*	*uniform*

Standard errors clustered by *Cultureef*. Asterisks denote significance levels (* = 0.10, ** = 0.05, *** = 0.01). Each cell presents a separate estimate from a *rdrobust* analysis.

Commonly, the *triangular* kernel function is advised to be employed as an optimal coefficient will be obtained when it is combined with MSE-optimal bandwidth [81]. As for the order of the local polynomial, local linear regressions are considered to be the our main estimates while adopting quadratic polynomial of the running variable as tests for robustness of our results [87].

Therefore, Columns (1)–(4) of Table 6 show the RD estimates based on MSE-optimal bandwidth selection along with first order polynomial and *triangular* kernel function, and the treatment effect of the normative policies on *Cultureis* is 1.223, which indicates the rate of the key words representing *Cultureis* rose by 1.223% significantly. Accordingly, Columns (5)–(8) of Table 6 present the estimator of the restrictive policies on *Cultureis* is 1.469, implying the rate of the key words representing *Cultureis* significantly increased by 1.469%. In summary, both the normative and restrictive policies impose a significant positive effect on *Cultureis*, which is in accord with H1 and H2.

As is displayed in the Columns (1)–(8) of Table 7, the RD estimates under distinct bandwidths, orders of polynomial and kernel functions appear to be entirely insignificant, which suggests that the frequency of the key words representing *Cultureif* hardly varies whether it is treated by the normative policies or restrictive policies, which lives up to the expectations of H3 and H4.

Next, the treatment effect of normative policies on *Culturees* is reported in all the Columns (1)–(8) of Table 8. Apparently, there are two cutoffs of the running variable, where the cutoff 1 was treated by the normative policies and the cutoff 2 actually by the encouraging policies. Although the statistical significance in the table manifests that both the normative and encouraging policies impact *Culturees* significantly, yet the coefficient of the cutoff 1 is significantly negative, reducing by 0.246%, while the cutoff 2 significantly positive, rising by 0.268%. Additionally, the temporal distance between the two cutoffs is around a period of 5 weeks, indicating a short-term effect of the normative policies, which is in agreement with H5. With regard to the treatment effect of restrictive policies on *Culturees* displayed in Table 9, Column (1), the RD estimator shows a significant reduction of the strength of *Culturees*, by 0.135%, which corresponds with H6.

Last, the treatment effects of the two regulatory policies on *Cultureef* are dissimilar (see Table 10). In detail, the normative policies do not take on a significant treatment on *Cultureef* in Columns (1)–(4), whereas the significant strengthening after the implementation of the restrictive policies is shown in Columns (5)–(8), which signifies that the intensity of externality-flexibility culture rose by 0.386% significantly. This regression result conforms to H7 and H8.

### 4.4 Robustness tests and validity checks

To avoid potential bias, seven approaches are recommended to test the robustness of RDiT estimates, like Donut Hole estimate, placebo tests and so forth [82]. Normally, there is a concern to perform RD estimates whether the results are sensitive to the length of bandwidth. As a consequence, in the previous statement, different times of MSE-optimal bandwidths, orders of polynomial and kernel functions have been displayed as part of our robustness checks. As is exhibited in Tables 6–10, approximately all the *rdrobust* and *rdms* regression results remain robust to the conditioned sets. Therefore, we cautiously come to the conclusion that the RD estimators have moderately successfully passed the bandwidth sensitivity test.

Further, in this subsection, two other effective robustness tests are applied: the placebo experiments and the Donut Hole approach.

For one thing, the placebo experiment is a fundamental and informative tool of robustness checks for RD. The rationale of placebo tests lies in there being more likely a real cutoff if the hypothetical cutoffs in placebo tests do not exist. Naturally, the statistical significance with the placebo outcome will cause the validity of the research design open to doubt [92]. Regarding our design for the placebo experiments, it is noteworthy that the means of arbitrarily assigning a cutoff cannot be employed directly because the newly hypothetical cutoff may use the values on either side of the original cutoff to perform an estimate, which may draw a conclusion of significance that would not have existed. Thus, in view of the method adopted by Akbulut-Yuksel et al. [87], we similarly specially concentrate on the observations from the pre-policy or post-policy period and obtain the estimators according to the local linear regression, manual bandwidth and *triangular* or *uniform* kernel function by pretending one threshold at the median value of the running variable to test the sensitivity of our cutoffs. It is smoothly calculated that the median value for the weeks 1–25 (the pre-policy period) is the 13th week, and for the weeks 26–50 (the post-policy period) it is the 38th week. With regard to the two cutoffs of the normative policies on *Culturees*, only the periods of weeks 1–25 and weeks 31–50 are tested, that is, the cutoff at week 13 and 41, owing to the relatively short time span of weeks 26–30. Moreover, due to the limited number of observations, we offer to set a larger bandwidth to include more data to test the estimates. In Table 11, all the estimators are statistically insignificant, which indicates that our original hypotheses (H1–H8) are more reliable and robust.

**Table 11:** The treatment effect of regulatory policies with the placebo experiments.

	Pre-policy placebo cutoff		Post-policy placebo cutoff	
	Cutoff at week 13	Cutoff at week 13	Cutoff at week 38	Cutoff at week 38
Normative policies on *Cultureis*	0.027 (0.075)	0.061 (0.084)	-0.073 (0.057)	-0.023 (0.082)
Restrictive policies on *Cultureis*	-0.165 (0.361)	-0.103 (0.327)	-0.284 (0.303)	-0.350 (0.305)
Normative policies on *Cultureif*	-0.007 (0.005)	-0.004 (0.006)	-0.003 (0.007)	-0.003 (0.007)
Restrictive policies on *Cultureif*	0.014 (0.010)	0.012 (0.009)	-0.003 (0.007)	-0.003 (0.007)
Restrictive policies on *Culturees*	0.025 (0.058)	0.062 (0.064)	0.000 (0.006)	0.000 (0.007)
Normative policies on *Cultureef*	0.031 (0.040)	0.010 (0.042)	0.012 (0.036)	0.018 (0.035)
Restrictive policies on *Cultureef*	-0.040 (0.031)	-0.024 (0.035)	0.068 (0.067)	0.096 (0.076)
			Cutoff at week 41	Cutoff at week 41
Normative policies on *Culturees*	-0.024 (0.048)	-0.033 (0.048)	0.035 (0.080)	0.055 (0.076)
Polynomial	Linear	Linear	Linear	Linear
Bandwidth	15	15	15	15
Kernel function	*triangular*	*uniform*	*triangular*	*uniform*

Cluster standard errors are adopted. Asterisks denote significance levels (* = 0.10, ** = 0.05, *** = 0.01). Each cell presents a separate estimate from a *rdrobust* analysis.

For another thing, as mentioned in the potential manipulation section, there is little possibility for off-campus training institutions to escape the treatment of policy change. But here we adopt the Donut Hole approach to further confirm the robustness of our results, which means eliminating the observations around the cutoff to avoid the potential manipulation to figure out whether the results are still robust. Meanwhile, the local linear polynomial, MSE-optimal bandwidth and *triangular* kernel function are employed. As is presented in Table 12, five different Donut Hole radii are employed: Column 2 (dropping week 26, when the policy change occurred), Column 3 (dropping weeks 25–26), Column 4 (dropping weeks 25–27), Column 5 (dropping weeks 24–27) and Column 6 (dropping weeks 24–28). In the meantime, the baseline RD estimator is provided to work as a reference point. As is shown in Table 12, although several observations around the cutoff are deleted, the new estimators and significance levels almost remain the same as those of the corresponding baseline RD, which implies little manipulation around the cutoff and robustness of the RD. Moreover, considering the multi-cutoffs of the normative policies on *Culturees*, the similar process of excluding observations around the cutoffs are applied. Similarly, the new estimators and significance levels in Table 13 almost keep pace with the original baseline RD estimates.

**Table 12:** The treatment effect of regulatory policies with the Donut Hole approach.

	Baseline RD estimator	Donut Hole Drop week 26	Donut Hole Drop weeks 25–26	Donut Hole Drop weeks 25–27	Donut Hole Drop weeks 24–27	Donut Hole Drop weeks 24–28
	(1)	(2)	(3)	(4)	(5)	(6)
Normative policies on *Cultureis*	1.223*** (0.127)	0.966*** (0.035)	1.072*** (0.087)	1.090*** (0.092)	1.243*** (0.047)	1.281*** (0.038)
Restrictive policies on *Cultureis*	1.469*** (0.110)	1.513*** (0.187)	1.568*** (0.200)	1.428*** (0.182)	1.478*** (0.158)	1.715*** (0.192)
Normative policies on *Cultureif*	-0.004 (0.008)	-0.009 (0.008)	-0.015 (0.011)	-0.011 (0.011)	0.004 (0.007)	0.001 (0.007)
Restrictive policies on *Cultureif*	-0.003 (0.002)	0.002 (0.004)	-0.002 (0.004)	0.005 (0.004)	-0.002 (0.004)	-0.002 (0.006)
Restrictive policies on *Culturees*	-0.135*** (0.021)	-0.137*** (0.023)	-0.179*** (0.047)	-0.180*** (0.047)	-0.229*** (0.070)	-0.229*** (0.070)
Normative policies on *Cultureef*	0.014 (0.025)	-0.027 (0.031)	-0.013 (0.038)	-0.048 (0.040)	-0.054 (0.063)	-0.058 (0.067)
Restrictive policies on *Cultureef*	0.386*** (0.105)	0.513*** (0.046)	0.525*** (0.047)	0.472*** (0.083)	0.496*** (0.085)	0.532*** (0.035)
Polynomial	Linear	Linear	Linear	Linear	Linear	Linear
Bandwidth	MSE	MSE	MSE	MSE	MSE	MSE
Kernel function	*triangular*	*triangular*	*triangular*	*triangular*	*triangular*	*triangular*

Cluster standard errors are adopted. Asterisks denote significance levels (* = 0.10, ** = 0.05, *** = 0.01). Each cell presents a separate estimate from a *rdrobust* analysis.

**Table 13:** The treatment effect of normative policies on *Culturees* with the Donut Hole approach.

	Baseline RD estimator at cutoff 1	Donut Hole Drop week 25	Donut Hole Drop weeks 24–26	Donut Hole Drop weeks 22–26	Baseline RD estimator at cutoff 2	Donut Hole Drop week 31	Donut Hole Drop weeks 30–32	Donut Hole Drop weeks 30–34
	(1)	(2)	(3)	(4)	(5)	(6)	(7)	(8)
Normative policies on *Culturees*	-0.246*** (0.002)	-0.169*** (0.002)	-0.286*** (0.054)	-0.383*** (0.029)	0.268*** (0.038)	0.342*** (0.028)	0.279*** (0.030)	0.212*** (0.021)
Polynomial	Linear	Linear	Linear	Linear	Linear	Linear	Linear	Linear
Bandwidth	MSE	MSE	MSE	MSE	MSE	MSE	MSE	MSE
Kernel function	*triangular*	*triangular*	*triangular*	*triangular*	*triangular*	*triangular*	*triangular*	*triangular*

Cluster standard errors are adopted. Asterisks denote significance levels (* = 0.10, ** = 0.05, *** = 0.01). Each cell presents a separate estimate from a *rdrobust* analysis.

## 5. Discussion

In this section, the aforementioned results of RD designs are given an in-depth discussion, and then the theoretical significance and the possible practical implications of this study are further explored.

According to Table 6, both the normative policies and restrictive policies have a statistically significant positive impact on the internality-stability culture (*Cultureis*) containing cultural elements like governance, laws, regulations and so forth, and the estimators of their treatment effects are similar. What should be pointed out, however, is that the policy backgrounds behind them are different. Ahead of the treatment effect of normative policies, encouraging policies played a role in the development of off-campus training institutions, while normative policies took the lead before the appearance of restrictive policies. In comparison, normative policies have higher regulatory requirements for off-campus training schools than encouraging policies, which indicates that restrictive policies have a stronger regulatory intensity on off-campus training institutions.

As is presented in Table 7, despite both the normative and restrictive policies having no significant correlation with the internality-flexibility culture (*Cultureif*), we can also see that in the database of China Digital Library, the reporting of various levels and types of newspapers and media on the internality-flexibility culture of private tutoring institutions is remarkably limited, with the mean values of 0.013% and 0.007% in Table 3 as well as 0.005% and 0.004% in Table 4, and the maximum value is only 0.035%, shown in Table 4, which is relatively and obviously low, compared with the values of other cultures. The potential reason behind it is that the key index indicators involved in *Cultureif*, such as authorization, empowerment, employee participation, team cooperation, and skill or employee training, are relatively private within the enterprises and are not easy to be discovered and reported by external personnel. Therefore, due to the comparatively small number of values reflected in this database, we hold a more cautious attitude towards the result that there is no statistically significant correlation between the regulatory policies and the internality-flexibility culture of enterprises.

From Table 8, it can be seen that the regulatory policies produced statistically significant positive or negative effects at the two different cutoffs. The first threshold reflects a significant weakening effect of normative policies on the externality-stability culture of enterprises (*Culturees*). Nevertheless, this weakening effect did not last long and *Culturees* was significantly strengthened after five weeks. At this point (the second threshold), it was actually the encouraging policies that began to play a strengthening role as the normative policies did not restrict the large-scale growth of off-campus training institutions. In addition, according to the RD estimators from Table 8, the intensity of *Culturees* after the second cutoff (after the encouraging policies) was almost at the same level as before the first cutoff (before the normative policies).

Table 9 displays that the restrictive policies imposed a statistically significant negative effect on the externality-stability culture of corporations (*Culturees*). Moreover, the weakening effect is extraordinarily strong, and after being subject to the restrictive policies, the intensity of the externality-stability culture is almost negligible. The cultural elements such as vision, prospects, financing, listing, development strategies, and missions have essentially been lost from the corporate culture.

Taking Table 10 into account, there is no statistically significant change in the externality-flexibility culture of corporations (*Cultureef*) after the normative policies while the restrictive policies are fairly distinct, which significantly enhanced the innovation, upgrading, iteration, transformation, and other similar elements of organizational culture. The possible reason is that restrictive policies limit the scale development and profitability level of enterprises. Thus, the original business of enterprises is greatly challenged and naturally the corporate culture must be innovated, upgraded, and transformed to adapt to the new operation environment.

The theoretical significance of this research mainly consists in three aspects. First, there are a load of determinants that contribute to the formation and evolution of organizational or corporate culture, as previous literature has mentioned, such as social institutions like collectivism [93], national culture [94,95], characteristics of a certain industry including market competition, customer demands, and societal anticipation [96], different characteristics across industries [97], leaders and founders’ creating and embedding culture [64], ownership structure type and firm size [13], and other factors like geographic location, the personalities of staff etc. [98]. Nevertheless, based on our research results and analyses, it has been found that regulatory policies can also have varying degrees of strengthening and weakening effects on different aspects of corporate culture, the significance of which lies in analyzing the formation and evolution of corporate culture from the perspective of policy treatment effects, and thus further filling the research gap of the extant theories and literature in the realm of corporate culture.

Second, organizational culture has often been measured by applying various questionnaire surveys, for instance, the OCAI [18], the DOCS [12], Hofstede’s adopting a three-phase design to determine the dimensions of organizational culture [66]. In this study, however, capitalizing on MAXQDA 18 software embedded with the advanced computer-assisted algorithms for tokenization, sentence segmentation and word frequency techniques, we make an attempt to carry out the content analysis to obtain data on four dimensions of corporate culture. Further, previous literature on measuring corporate culture through content analysis has identified various vehicles, including annual reports of listed firms [49–51], 10-K filings of listed firms [52–55], earnings call transcripts [56,57], statements about core values on corporate official websites [58,59], employee reviews [60] and the like. It is worth noting that, although media coverage as a medium of content is also considered to be used for measuring corporate culture, yet media coverage, in extant literature, is mainly utilized for sentiment analysis [35–39] and other areas, like images and portrayals of famous people [99], and national cultural traits [100]. Consequently, this study extends the scope of content analysis by adopting the approach of analyzing news stories to measure organizational culture.

Third, in the extant literature applying corporate culture as the dependent variable, certain approaches of data analysis are turned to account, like ordinary least squares [13–15], structural equation modelling [95], qualitative analysis [64,96,98], Q-sort method [97]. In this study, nonetheless, the quasi-experimental sharp RD, regression discontinuity in time (RDiT), and RD with multiple cutoffs designs are employed, which further advances the research methods of corporate culture. Moreover, RD designs have been applied in various fields of social sciences including economics, policy science, education, labor markets, health, environment, and so forth [78,80]. We, nevertheless, bring the RD designs to bear on evaluating the effect of policy treatment on corporate culture, which further increases the range of applications of RD designs.

In addition, this research also has practical value. For one thing, over the past 40 years of reform and opening-up in China, due to the government’s encouraging and supportive policies towards enterprises in various aspects, a wealth of enterprises has focused more on market competition, corporate leaders, and internal management in shaping and evolving corporate culture, which, to some degree, has led to an oversight of the significant impact that government regulatory policies can have on corporate culture. In view of the encounter of China’s off-campus training institutions, it would be wiser that companies should pay greater attention to regulatory policies and assess their potential impact on the enterprises since the strengthening and weakening effects on corporate culture imposed by different types of policies cannot be ignored, which can deeply affect the operations and development of the corporations. For another thing, as for policy recommendations, due to the guiding nature of regulatory policies—assisting enterprises in understanding how to adjust their corporate culture to comply with regulations—the formulation and imposition of explicit industry standards and specific operational norms can be executed by the government, which contributes to enhancing the normativeness of corporations and thus shaping a beneficial corporate culture for stakeholders. Further, if necessary, restrictive policies limiting the scale and profitability of businesses can be enacted and implemented, thereby reducing the damage of externality-stability culture (keywords like financing, capital and public listing) including industry overexpansion, excessive consumption of societal resources, public anxiety etc. Notably, it is necessary that advance assessment of the potential economic consequences resulting from varying degrees of regulatory policies should be conducted, since certain regulatory policies, such as restrictive ones, may bring about disruptive changes to corporate culture. This could render some businesses incapable of promptly adjusting to the substantial impact from these industry-specific regulatory policies, thus leading to widespread financial losses or even compelling them to cease operations.

## 6. Conclusion

For a long time, as a factor outside the market, the impact of industry-level regulatory policies on corporate culture has not been fully paid attention to and studied. However, based on two major experiences of China’s off-campus training industry being regulated, we have found that they have had a dramatic impact on the companies and their corporate culture has also undergone profound changes. Therefore, combined with the techniques of text analysis and the organizational culture survey of off-campus training institutions in China adapted from the Denison Organizational Culture Survey (DOCS), this paper collects the content of 4,000 news reports (about 4 million words) to obtain the data on corporate culture. Then, the RD designs are used to infer the treatment effect of policies on corporate culture, and finally the following research conclusions are drawn.

To start with, both the normative policies and the restrictive policies have a significant positive impact on the internality-stability culture (*Cultureis*), whose intensity rose by 1.223% and 1.469% respectively. By comparison, though the estimators of the two policy changes are similar, yet the degree of the impact of the restrictive policies is greater.

Next, the RDiT estimates display no statistically significant variance in the internality-flexibility culture (*Cultureif*) when treated by both the two industry-level regulatory policies. Nevertheless, on account of the cultural components regarding *Cultureif* being comparatively less reported on the news media, we hold a more cautious attitude towards the statistically insignificant effect of the regulatory policies.

Besides, there exists an observable dissimilarity between the two regulatory policies in the externality-stability culture (*Culturees*). As for the normative policies, the intensity of the externality-stability culture significantly reduced by 0.246% for 5 weeks at the first cutoff and then significantly rose by 0.268% at the second, which almost returned to the level before the policy change. These two thresholds present a temporary significant impact from the normative policies on *Culturees*_._ In comparison with the two cutoffs, the restrictive policies only left a significant negative effect on the externality-stability culture, the estimator of which is -0.135%. Remarkably, the cultural elements including vision, prospects, financing, listing, development strategies, and missions have almost vanished from the organizational culture.

Finally, the externality-flexibility culture (*Cultureef*) was influenced significantly and positively by the restrictive policies, whose estimator of treatment effect is 0.386%, while the normative policies did not play a statistically significant role in *Cultureef* according to the RDiT estimates.

On the whole, industry-level regulatory policies may have a significant positive or negative impact on the different dimensions of organizational culture. We caution business operators to pay more attention to regulatory policies and assess their potential treatment effect on organizational culture to better prepare for the management and development of their businesses. With regard to policymakers, the formulation and implementation of industry regulatory policies hold instructive significance for all walks of life, which aids enterprises in adjusting their culture to enhance operational compliance and curbing hazards from the dimension of externality-stability culture as well. Nevertheless, governments had better perform an early assessment of the impact intensity of regulatory policies on corporate culture to ensure that potential financial losses in regulated industries are controlled within anticipated boundaries.

## Supporting information

S1 Appendix Organizational culture survey of off-campus training institutions in China. (DOCX)

S1 Dataset The normative policies. (DTA)

S2 Dataset The restrictive policies. (DTA)

S1 File A Do file running in the Stata 16 version. (DO)
