# Supplementary material for: The role of regulatory policies in organizational culture: Insights from the education industry
Source: PLoS One. 2024 May 15;19(5):e0299848. doi: 10.1371/journal.pone.0299848 (PMC11095740; doi:10.1371/journal.pone.0299848)
Supplement: S1 Appendix — (DOCX) [file pone.0299848.s001.docx]

**S1 Appendix. Organizational culture survey of off-campus training institutions in China**

(Adapted from the Denison Organizational Culture Survey)

| Trait | Index | Bag of key words |
| --- | --- | --- |
| The internality-stability culture (Consistency) | Core values | forbid, investigate, supervise, regulate, regulation, oversee, inspect, rectify, reorganize, remediate, govern, law, stipulation, norm, rule, legality, lawfulness, approval, double reduction, constitution |
|  | Agreement | achieve “win-win” solutions, reach agreement, communicate |
|  | Coordination and integration | coordinate across departments, work with someone from another department, coordination |
| The internality-flexibility culture (Involvement) | Empowerment | empowerment, authorization, job involvement, staff participation, shared information, sense of ownership |
|  | Team orientation | team orientation, cooperation across departments, teamwork |
|  | Capability development | autonomous decision-making, improve ability, staff training, skill training, teacher training, training plan |
| The externality-stability culture (Mission) | Vision | vision, foresight, prospect, long-term viewpoint, forethought, prosperity, booming |
|  | Goals and objectives | goals, objectives, financing, listing, capital |
|  | Strategic direction and intent | strategy, direction, mission |
| The externality-flexibility culture (Adaptivity) | Organizational learning | innovate, take risks, create, refresh, invent, renew, update, restart, upgrade, iterate |
|  | Customer focus | customer focus, customer-centered, customer need, customer experience, customer service |
|  | Creating change | reform, ameliorate, remold, adapt, cancel, job transfer, change profession, transform, slash, eliminate, outlaw |
